# Toward precision detection of pyrazinamide resistance: critical concentration assessment and rapid molecular method validation

**DOI:** 10.3389/fmicb.2026.1828630

**Published:** 2026-05-25

**Authors:** Yanfeng Zhao, Lili Tian, Nenhan Wang, Hao Chen, Shuangshuang Chen, Lan Yu, Mengdi Pang, Beichuan Ding, Jie Li, Chuanyou Li, Xiaowei Dai

**Affiliations:** 1 Beijing Center for Disease Prevention and Control, Beijing, China; 2 School of Public Health, Capital Medical University, Beijing, China

**Keywords:** critical concentration, MeltPro MTB/PZA assay, minimum inhibitory concentration, PncA gene mutations, pyrazinamide, rifampicin-resistant tuberculosis, whole-genome sequencing

## Abstract

This study evaluated broth microdilution (BMD) and fluorescence PCR melting curve analysis (MeltPro MTB/PZA, targeting *pncA* mutations) for detecting pyrazinamide (PZA) resistance-associated mutations in rifampicin-resistant tuberculosis (RR-TB). PZA susceptibility was assessed in RR-TB isolates from patients at TB prevention and control institutions and at designated hospitals in Beijing. Whole-genome sequencing (WGS) was used as a genotypic comparator to assess the agreement of BMD at critical concentrations (CCs) of 100 and 200 μg/mL and of the MeltPro assay. BMD showed resistance rates of 63.6% (70/110) at 100 μg/mL and 45.5% (50/110) at 200 μg/mL. Relative to WGS-defined genotypes (wild-type vs. mutation), BMD at 100 μg/mL showed 96.1% agreement for isolates with mutations and 64.4% for wild-type (*κ* = 0.590); at 200 μg/mL, agreement was 92.2% for mutations and 94.9% for wild-type (*κ* = 0.872). The MeltPro assay showed 89.7% agreement for isolates with mutations and 98.4% for wild-type (*κ* = 0.882). These findings indicate that using BMD at 200 μg/mL as the critical concentration achieves closer agreement with WGS-defined genotypes than 100 μg/mL, and that the MeltPro MTB/PZA assay shows high concordance with WGS for rapid detection of pncA mutations in RR-TB isolates. Further validation using phenotypic reference methods and clinical outcomes is warranted.

## 1 Introduction

Tuberculosis (TB) remains a leading global infectious cause of death, with an estimated 10.7 million new cases and 1.23 million deaths in 2024. Multidrug-resistant or rifampicin-resistant TB (MDR/RR-TB) accounts for 3.2% of new cases and 16% among previously treated cases worldwide (World Health Organization, 2025). Pyrazinamide (PZA), widely used in both first- and second-line regimens, demonstrates unique sterilizing activity against semi-dormant bacilli. However, PZA resistance has been increasing globally, with particularly sharp rises observed in China. Between 2000 and 2010, resistance rates in non-MDR isolates climbed from 8.0 to 11.3%, while in MDR-TB isolates they increased from 29.6 to 50% (Pang et al., 2017). Understanding PZA susceptibility as early as possible is crucial for guiding the selection and design of effective regimens.

Current phenotypic drug susceptibility testing (pDST) for PZA faces significant limitations. The bactericidal activity of PZA depends on its conversion to pyrazinoic acid (POA) under acidic conditions (pH ~ 5.5). However, these acidic conditions, required for PZA activation, concurrently inhibit *Mycobacterium tuberculosis (M. tuberculosis)* growth, rendering the Lowenstein-Jensen proportion method unreliable for PZA susceptibility testing (Zhang et al., 2002). The gold standard for resistance detection is culture-based drug susceptibility testing using BACTEC MGIT 960 system with PZA medium. Nevertheless, this assay has been associated with false-resistant results due to alkalization of the medium caused by a high inoculum size or the presence of bovine serum albumin. Another limitation is the long turnaround time, as the test requires a primary culture and is often performed on a secondary culture (Havlicek et al., 2019). The broth microdilution (BMD) method can provide not only qualitative susceptibility results but also the minimum inhibitory concentration (MIC), which indicates the degree of drug resistance. However, because it requires an acidic environment, PZA cannot be included on the same culture plate as other drugs (World Health Organization, 2022c). This technical limitation has been addressed in commercially available multi-drug MIC plate designs. Recent research has demonstrated the feasibility of cultivating PZA alongside other first-line drugs on a single, standardized plate, effectively overcoming this hurdle (Pang et al., 2024). Despite this progress, a major challenge remains. Neither the WHO nor the CLSI has recommended a PZA-specific critical concentration (CC) for the BMD method, complicating standardized resistance interpretation. This lack of a consensus CC represents a key hurdle to the wider adoption of BMD for PZA susceptibility testing. The 2023 WHO guidelines prioritize whole-genome sequencing (WGS) as the reference method for detecting *pncA* mutations (World Health Organization, 2023a). WGS can provide near-complete information by capturing the entire genetic repertoire of a given clinical *M. tuberculosis* strain (Miotto et al., 2017). However, its cumbersome nature, cost, and technical accessibility limit its scalability in high-burden, resource-limited settings (Hu et al., 2024). Alternative molecular methods, such as targeted *pncA* sequencing (e.g., MeltPro MTB/PZA), demonstrate 94.4% accuracy compared to WGS while being more cost-effective and rapid (He et al., 2025).

This study, focusing on RR-TB isolates, aimed to assess the agreement of BMD at different critical concentrations with WGS-defined genotypes and to evaluate the concordance of the MeltPro MTB/PZA assay with WGS. The goal was to provide genotype-informed reference data to support PZA susceptibility testing in RR-TB patients.

## 2 Materials and methods

### 2.1 Subculture of *Mycobacterium tuberculosis* clinical isolates

Clinical isolates of *M. tuberculosis* were retrieved from storage at −80 °C and thawed at room temperature. A 100 μL aliquot of each bacterial suspension was inoculated onto neutral Löwenstein–Jensen (L-J) medium (Celnovte Biotechnology Co., Zhengzhou, China). The culture tubes were gently rotated to ensure even distribution of the inoculum over the slanted surface. Subsequently, the tubes were incubated at 37 °C (Memmert IPP260 incubator, Schwabach, Germany) and observed weekly for colony growth.

### 2.2 BMD susceptibility testing

PZA susceptibility testing was performed using the *Mycobacterium tuberculosis* Drug Susceptibility MIC Plate (BASO Diagnostics Inc., Zhuhai, China; Batch No. 23091501 and 23,091,502). An English layout diagram (plate map) detailing the drug arrangement is provided as Supplementary Figure S1. Several colonies in the logarithmic growth phase were selected to prepare a bacterial suspension equivalent to a 1.0 McFarland standard, which was then diluted 1:100 with the kit-provided PZA-specific medium (pH = 6.0 ± 0.3). The pH of the medium was verified before and after incubation using pH test strips to ensure stability; results are provided in Supplementary Figure S2. Using an auto-inoculator (BSJ-9612, BASO Diagnostics Inc., Zhuhai, China), 100 μL aliquots were dispensed into each well. The sealed plates were incubated at 37 °C for 10–14 days, and the MIC was interpreted using a dedicated analysis system (BSP-TB96, BASO Diagnostics Inc.). All experimental steps were performed in strict accordance with the manufacturer’s instructions.

### 2.3 Minimum inhibitory concentration (MIC) determination

The *M. tuberculosis* H37Rv strain (ATCC 27294) was used as the quality control strain for each batch of experiments. A batch was considered valid only if the bacterial growth was observed in the positive control well (drug-free). If the positive control wells showed no growth or signs of contamination, the experiments were repeated. For the PZA-specific controls, growth was present in the PZA-positive control wells (50 and 100%), confirming the validity of the test conditions. According to the product instructions, the MIC of PZA was defined as the lowest drug concentration that inhibited visible bacterial growth relative to the 50% positive control. The concentration range of PZA in the microplate was 25–800 μg/mL. For result interpretation in this study, we applied critical concentrations (CC) of both 100 μg/mL and 200 μg/mL.

### 2.4 DNA extraction

Bacterial colonies were harvested during the logarithmic growth phase and transferred to 1.5 mL microcentrifuge tubes containing 1 mL of sterile normal saline. Then, the suspensions were heat-inactivated at 99 °C for 10 min and subsequently centrifuged at 12,000 × g for 2 min. The resulting supernatant was used as the template for nucleic acid amplification.

### 2.5 Meltpro MTB/PZA assay for rapid detection of PZA resistance in *Mycobacterium tuberculosis*

DNA was extracted from the colonies using an automated system (LabAid-824S; Zeesan Biotech, Xiamen, China). The MeltPro MTB/PZA assay (MeltPro® MTB/PZA Test Kit; Zeesan Biotech, Xiamen, China; Batch No. 23081501) was then performed according to the manufacturer’s instructions, providing a fully automated process. Briefly, the assay used PCR and melting curve analysis methods in four separate reaction tubes. It targeted mutations within the 561-bp *pncA* gene and its promoter region (positions −16 to −1) to determine PZA resistance.

### 2.6 Whole genome sequencing and bioinformatics analysis

Extracted genomic DNA was subjected to WGS on an Illumina NovaSeq next-generation sequencing platform. Library preparation was performed using 100–500 ng of bacterial DNA with the Illumina DNA Prep Kit (20,060,059, Illumina, San Diego, CA, USA) according to the manufacturer’s protocol. Briefly, DNA was fragmented using transposase-mediated tagmentation, followed by the addition of adapter sequences. The resulting fragments were size-selected for an optimal insert length of approximately 300–350 bp, then enriched and quantified. Sequencing was conducted on the Illumina NovaSeq platform using the NovaSeq X Series 1.5B Reagent Kit (300 cycles) (20,104,705, Illumina, San Diego, CA, USA), generating paired-end reads with an average length of 302 bp. For bioinformatics analysis, raw read quality was assessed using the fastp tool with default parameters. The BWA aligner was used to map the sequences to the *Mycobacterium tuberculosis* reference genome H37Rv (GenBank accession no. NC_000962.3). Subsequently, the TB-Profiler tool was employed to determine the spoligotype and to identify mutations associated with resistance to anti-tuberculosis drugs. A summary table, including the accession number for each genome, the corresponding PZA MIC values, and the results of the MeltPro MTB/PZA assay, is provided in Supplementary Table S1. Large deletions detected by TB-Profiler were manually verified using Integrative Genomics Viewer (IGV) (see Supplementary Table S2).

### 2.7 Interpretation criteria for drug resistance-associated mutations

According to the WHO guideline “Catalogue of mutations in *Mycobacterium tuberculosis* complex and their association with drug resistance” (Second Edition) (World Health Organization, 2023b), the association between detected gene mutations and anti-tuberculosis drug resistance was classified into one of five established categories:

- Group 1: Associated with resistance.
- Group 2: Associated with resistance – interim.
- Group 3: Uncertain significance.
- Group 4: Not associated with resistance – interim.
- Group 5: Not associated with resistance.

Groups 1 and 2 variants should be interpreted as markers of clinically relevant phenotypic resistance, whereas Groups 4 and 5 variants are not markers of resistance. The role of Group 3 mutations remains uncertain from the available evidence. For mutations not included in the abridged variant classification for PZA, grouping was performed according to the additional grading rules provided in the same guideline.

### 2.8 Statistical analysis

Statistical analysis was performed using SPSS software (version 19.0; IBM, Armonk, NY, USA). Categorical data are presented as rates or proportions. WGS results served as a genotypic comparator for assessing the agreement (including the Kappa value) of the BMD method and the MeltPro MTB/PZA assay with WGS-defined genotypes. The strength of agreement based on the Kappa statistic was interpreted as follows: 0.40–0.59, Weak; 0.60–0.79, Moderate; 0.80–0.90, Strong; and above 0.90, Almost Perfect (McHugh, 2012). Ninety-five percent confidence intervals (95% CIs) were calculated using the online tool available at http://vassarstats.net/index.html.

## 3 Results

### 3.1 Study selection

**Figure 1:**
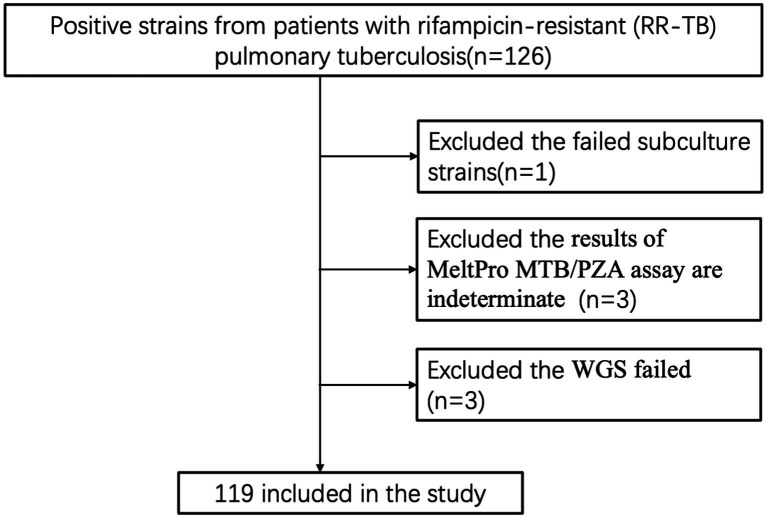
Flow chart of sample collection. Graph show number of isolates included in the study.

The study population included RR-TB isolates, as identified by the solid proportion method, from 126 eligible TB patients. These patients were treated in TB prevention and control institutions and designated hospitals in Beijing between January and December 2009. In total, 119 isolates were selected for BMD, MeltPro MTB/PZA assay, and WGS to detect PZA resistance. The remaining 5 isolates were excluded because they were subculture negative, had indeterminate MeltPro MTB/PZA assay results, or had insufficient DNA quantity and quality (Figure 1).

### 3.2 Spectrum of PZA MICs and its correlation with *pncA* mutation categories

**Figure 2:**
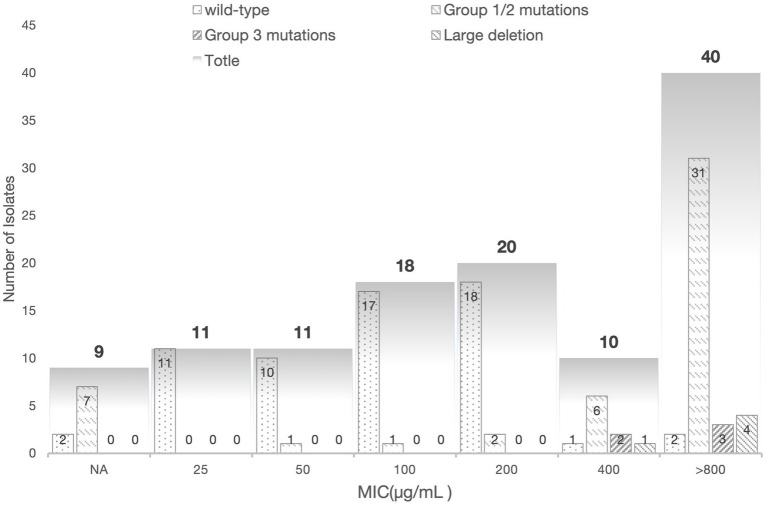
Distribution of *pncA* genotype categories across pyrazinamide MIC intervals. not available (NA). Grouped column chart showing the number of isolates for each *pncA* genotype category, clustered within their corresponding pyrazinamide MIC interval. MIC intervals are: NA, 25, 50, 100, 200, 400, and >800 μg/mL. Genotype categories are: wild-type, Group 1/2 mutations (associated with resistance/interim resistance), Group 3 mutations (uncertain significance), and large deletions.

Among the 119 RR-TB isolates, 9 (7.6%) showed no growth in the PZA positive control wells (confirmed by repeated tests) and were excluded from subsequent resistance rate calculations. The PZA MIC distribution for the remaining 110 isolates was as follows: 11 isolates (9.2%) each with MICs of 25 μg/mL and 50 μg/mL, 18 (15.1%) with 100 μg/mL, 20 (16.8%) with 200 μg/mL, 10 (8.4%) with 400 μg/mL, and 40 (33.6%) with an MIC > 800 μg/mL. Using CCs of 100 μg/mL and 200 μg/mL, the PZA resistance rates were 63.6% (70/110) and 45.5% (50/110), respectively. The distribution of *pncA* genotypes among PZA-resistant isolates across MIC intervals is shown in Figure 2. Among the 119 isolates, the wild-type genotype was the most prevalent (61 isolates, 51.3%), with the majority concentrated at MIC <200 μg/mL (58/69, 84.1%). Group 1/2 mutations were the next most common (48 isolates, 40.3%), and were particularly prominent at higher concentrations, accounting for the majority of isolates with MIC >800 μg/mL (31/40, 77.5%). Group 3 mutations were less frequent (5 isolates, 4.2%), and were primarily concentrated at MIC 400 μg/mL (2/5, 40.0%) and MIC >800 μg/mL (3/5, 60.0%). Large deletion genotypes (5 isolates, 4.2%) were observed mainly at the highest MIC (>800 μg/mL), accounting for 4 of these isolates (4/5, 80.0%).

### 3.3 Detection of the Meltpro MTB/PZA assay

Among 119 RR-TB isolates, 53 (44.5%) were resistant to PZA. Forty-two isolates had single-channel mutations (18 in Mix A, 13 in Mix B, 11 in Mix C), and 11 isolates had multiple-channel mutations (4 in Mix A and Mix B, 4 in Mix A and Mix C, 3 in Mix A, Mix B and Mix C). The mutation frequencies of each channel are provided in Supplementary Figure S3.

### 3.4 Determination of WGS PZA resistance mutation

WGS-based lineage analysis revealed that among the 119 RR-TB isolates, 113 (95.0%) belonged to Lineage 2 (East Asian/Beijing lineage) and the remaining 6 (5.0%) to Lineage 4 (Euro-American lineage). Detailed lineage information is provided in Supplementary Table S1. Further analysis of the WGS data showed that 58 of the 119 RR-TB isolates harbored mutations in PZA resistance-associated genes, yielding a mutation rate of 48.7% (58/119). All mutations were found in the *pncA* gene. The mutations were predominantly single nucleotide variants, accounting for 60.3% (35/58), while multi-nucleotide variants accounted for 6.9% (4/58). No synonymous mutations were detected. A total of 49 distinct mutation types were identified. The most frequent was the A → G transition at the promoter position −11, found in 4 isolates. Among these 4 isolates, 3 also carried mutations at other sites. The second and third most common mutations were T → C at codon 281 (Phe94Ser) and A → C at codon 226 (Thr76Pro), each detected in 3 isolates. Small deletion mutations were found in 7 isolates, insertion mutations in 5 isolates, and duplication mutations in 2 isolates. Given that two independent runs of TB-Profiler showed discrepancies in calling large deletions, we manually inspected the aligned sequences using the IGV to confirm the presence of these large deletions. Specifically, the first analysis identified large deletions in 5 isolates, while the second analysis reported “-” for the same isolates. Upon re-examination, the presence of large deletions was confirmed (IGV snapshots are provided in Supplementary Table S2). In all these cases, the indels occurred independently and were not found in combination with other types of *pncA* mutations (Table 1). Comparison of *pncA* structural mutations with PZA MIC values revealed that all five isolates harboring large deletions exhibited MICs ≥400 μg/mL, with the majority (4/5) > 800 μg/mL. Among isolates with frameshift mutations resulting from small indels (n = 14), the majority (8/14) showed MICs ≥400 μg/mL, while five isolates exhibited no growth in the positive control wells (NA). Four mutation types(456delC, 495delC, 389_390insA, and 520_521insT) were each identified in two independent isolates. No phenotypic heterogeneity was observed among isolates harboring the same mutation. Detailed data are provided in Supplementary Table S3.

**Table 1:** Mutation analysis of *pncA* gene in 119 RR-TB isolates.

*pncA* variant category	Site and nucleic change	Amino acid change	No. of isolates	WHO confidence grade
No variant detected	-	-	61	
Single nucleotide variant			35	
	−11A → G	Promoter	1	Group 1
	14 T → C	Ile5Thr	1	Group 3
	20 T → C	Val7Ala	1	Group 2
	80 T → A	Leu27Gln	1	Group 3
	104 T → C	Leu35Pro	2	Group 2
	137C → A	Ala46Glu	1	Group 2
	146A → C	Asp49Ala	1	Group 1
	151C → T	His51Tyr	2	Group 1
	169C → T	His57Tyr	1	Group 1
	175 T → C	Ser59Pro	1	Group 1
	185C → T	Pro62Leu	1	Group 1
	188A → C	Asp63Ala	1	Group 1
	199 T → C	Ser67Pro	1	Group 1
	200C → A	Ser67*	1	Group 2
	204G → A	Trp68*	1	Group 1
	206C → G	Pro69Arg	1	Group 3
	226A → C	Thr76Pro	2	Group 1
	241 T → G	Phe81Val	1	Group 3
	254 T → C	Leu85Pro	1	Group 1
	281 T → C	Phe94Ser	3	Group 2
	287A → G	Lys96Arg	1	Group 1
	404C → A	Thr135Asn	1	Group 2
	407A → G	Asp136Gly	2	Group 1
	416 T → C	Val139Ala	1	Group 1
	416 T → G	Val139Gly	1	Group 1
	512C → A	Ala171Glu	1	Group 2
	524 T → C	Met175Thr	1	Group 2
	538G → T	Val180Phe	1	Group 1
	539 T → G	Va180Gly	1	Group 1
Multi-nucleotide variant			4	
	−11A → G 139 A → G	Promoter Thr47Ala	1	Group 1 Group 1
	−11 A → G 460 A → G	Promoter Arg154Gly	1	Group 1 Group 1
	226 A → C 356 G → C	Thr76Pro Trp119Ser	1	Group 1 Group 3^a^
	−11 A → G 22 G → A 199 T → C 502 A → C	Promoter Asp8Asn Ser67Pro Thr168Pro	1	Group 1 Group 1 Group 1 Group 2
Deletion			7	Group 2
	116delC	Frameshift	1	
	366delA	Frameshift	1	
	399delT	Frameshift	1	
	456delC	Frameshift	2	
	495delC	Frameshift	2	
Insertion/duplication			7	Group 1^b^
	16_17insC	Frameshift	1	
	389_390insA	Frameshift	2	
	520_521insT	Frameshift	2	
	284dupA	Frameshift	1	
	409dupC	Frameshift	1	
Large deletion			5	-
	-270_*2851del ^b^	Gene disruption	1	
	277_427del^b^	Gene disruption	1	
	291_*5011del ^b^	Gene disruption	1	
	371_*857 del ^b^	Gene disruption	1	
	515_*66 del ^b^	Gene disruption	1	

Associated with resistance (Group 1); Associated with resistance–interim (Group 2); Uncertain significance (Group 3); Results from the second TB-Profiler analysis (−); Additional grading rules applied for final confidence grading (^a^); Data and grouping from the initial TB-Profiler analysis (^b^). *Stop codon.

### 3.5 Agreement of BMD and MeltPro MTB/PZA assay with WGS-defined genotypes

Using the WGS-based binary classification (Wild-type vs. Mutation) as the genotypic reference, we compared the results of BMD (at CCs of 100 μg/mL and 200 μg/mL) and the MeltPro MTB/PZA assay. A total of 110 isolates tested by BMD and 119 isolates tested by MeltPro were analyzed. BMD at 100 μg/mL showed agreement of 96.1% for isolates with WGS-identified mutations and 64.4% for wild-type isolates [positive predictive value (PPV) = 70.0%, negative predictive value (NPV) = 95.0%, *κ* = 0.590]. At 200 μg/mL, the agreement was 92.2% for isolates with mutations and 94.9% for wild-type isolates (PPV = 94.0%, NPV = 93.3%, *κ* = 0.872). The MeltPro assay showed agreement of 89.7% for isolates with WGS-identified mutations and 98.4% for wild-type isolates (PPV = 98.1%, NPV = 90.9%, *κ* = 0.882) (Table 2).

**Table 2:** Agreement of BMD and MeltPro MTB/PZA with WGS-defined genotypes.

	WGS		Agreement for mutations (95%CI, %)	Agreement for wild-type (95%CI, %)	PPV (95%CI, %)	NPV (95%CI, %)	Kappa value
Method/category	Wild-type	Mutation					
BMD (MIC = 100 μg/mL)			96.1 (86.8–98.9)	64.4 (51.7–75.4)	70.0 (58.5–79.4)	95.0 (83.5–98.6)	0.590
Sensitive	38	2					
Resistant	21	49					
Total	59	51					
BMD (MIC = 200 μg/mL)			92.2 (81.5–96.9)	94.9 (86.1–98.3)	94.0 (83.8–97.9)	93.3 (84.1–97.4)	0.872
Sensitive	56	4					
Resistant	3	47					
Total	59	51					
MeltPro MTB/PZA assay			89.7 (79.2–95.2)	98.4 (91.3–99.7)	98.1 (90.1–99.7)	90.9 (81.6–95.8)	0.882
Wild-type	60	6					
Mutation	1	52					
Total	61	58					

PPV, positive predictive value; NPV, negative predictive value; CI, confidence interval. Agreement is calculated relative to WGS-defined binary classification (wild-type vs. mutation) as a genotypic comparator. Isolates with WGS-identified mutations are shown as “mutation”; isolates with wild-type genotypes are shown as “wild-type”.

## 4 Discussion

According to WHO estimates, 390,000 people developed MDR/RR-TB globally in 2024, with China reporting 28,000 cases (World Health Organization, 2025). PZA is an important component of the standard first-line regimen for TB (World Health Organization, 2022a) and is also included in WHO consolidated guidelines for treating multidrug-resistant (MDR) and extensively drug-resistant TB (XDR-TB) (World Health Organization, 2022b). Recent studies have highlighted the growing challenge of PZA resistance in MDR-TB cases. A 2023 meta-analysis systematically evaluated this issue by estimating the global weighted pooled resistance (WPR) rate of PZA across WHO regions. Using STATA software for statistical analysis, the authors reported a PZA WPR of 57% (95% CI: 48–65%) in MDR-TB isolates (Wang et al., 2023). Moreover, after adding PZA to the treatment regimen, the treatment failure rates were twice as high in patients with PZA-resistant MDR-TB compared to those with PZA-sensitive MDR-TB (Bastos et al., 2014). Timely PZA susceptibility testing for MDR-TB isolates is essential for designing effective regimens and preventing the emergence of extensively drug-resistant strains. Several studies using the MGIT 960 system have reported PZA resistance rates in MDR-TB. These rates range between 41.01 and 57.7% (Xia et al., 2015; Jonmalung et al., 2010; Mphahlele et al., 2008; Ando et al., 2010; Wang et al., 2025; Gu et al., 2016). In this study, the BMD method was used to analyze the MIC distribution of PZA resistance among RR-TB isolates. Consistent with the requirements for PZA activation, the testing medium was maintained under acidic conditions. Post-incubation pH measurements confirmed that it remained acidic throughout the assay, thereby supporting the validity of the susceptibility results. At a CC of 100 μg/mL, the PZA resistance rate was 63.6%, while at 200 μg/mL, the resistance rate decreased to 45.5%.

Our analysis of *pncA* genotype categories across PZA MIC intervals revealed that wild-type isolates were predominantly concentrated at MICs <200 μg/mL. This is consistent with the expected phenotypic profile of PZA-susceptible or low-level resistant isolates. This also suggests that different phenotypic resistance interpretations may arise depending on the critical concentration used. Group 1/2 mutations were mainly found in isolates with MICs > 400 μg/mL, and particularly at MIC > 800 μg/mL, where they accounted for 77.5% (31/40) of the isolates. This further supports the role of Group 1/2 mutations in conferring PZA resistance, as recognized in the WHO catalogue. Notably, when using 200 μg/mL as the critical concentration (i.e., isolates with MIC < 200 μg/mL were classified as susceptible), only one isolate was classified as susceptible. This isolate carried a definitive Group 1/2 resistance mutation (pncA_p. Thr135Asn). This suggests that this mutation may be associated with low-level PZA resistance. Raising the breakpoint from 100 μg/mL to 200 μg/mL can exclude such low-level resistant mutants from being misclassified as resistant, thereby reducing false positives. However, this comes at the cost of potentially missing clinically relevant low-level resistance. In addition, all five isolates carrying Group 3 mutations exhibited MICs > 400 μg/mL. This provides evidence that such variants may indeed contribute to a resistant phenotype. These findings offer a clue toward resolving their “uncertain significance” in the WHO classification. Large deletion category were almost exclusively observed at MIC > 800 μg/mL, underscoring the substantial impact of structural gene disruption on the level of resistance. The WHO second-edition catalogue mentions an additional grading criterion, though it was not endorsed in that edition. This criterion proposed that any coding mutation in *pncA* (excluding silent mutations and Group 4 or 5 mutations) detected in genotypically rifampicin-resistant isolates could be regarded as a valid marker of PZA resistance. Our findings lend support to this proposed criterion. From a clinical perspective, the clustering of Group 1/2 mutations and large deletions at MIC > 800 μg/mL reinforces the importance of early genotypic detection to guide effective regimen selection. This is especially critical in RR-TB cases, where PZA remains a cornerstone drug.

To determine the optimal critical concentration for PZA resistance detection, we performed WGS on all RR-TB isolates in our cohort. The results demonstrated 93.6% concordance between sequencing data and the 200 μg/mL CC, with a Kappa value of 0.872 indicating excellent agreement. These findings indicate that using 200 μg/mL as the BMD critical concentration effectively reduces false-positive results for PZA resistance. From a clinical decision-making perspective, adjusting the critical concentration from 100 μg/mL to 200 μg/mL has two practical impacts. First, it would reclassify approximately 18% of RR-TB patients in this cohort from PZA-resistant to PZA-susceptible (resistance rate changing from 63.6 to 45.5%). This change would prevent these patients from being unnecessarily denied PZA, thereby preserving its clinical utility. Second, this adjustment carries the risk of misclassifying rare low-level resistant mutants (e.g., *pncA*_p. Thr135Asn) as susceptible. However, in our cohort, this occurred in only one isolate and such low-level resistance may still be overcome by standard PZA dosing given its pH-dependent activity. Therefore, we propose that using 200 μg/mL as the BMD critical concentration optimizes the balance by minimizing false-positive resistance calls without materially compromising the detection of clinically meaningful resistance.

PZA resistance in *M. tuberculosis* is acquired primarily through mutations in the *pncA* gene. These mutations either diminish the expression of pyrazinamidase/nicotinamidase (PZase) (Lamont et al., 2020) or reduce its enzymatic activity (Junaid et al., 2019; Khan et al., 2019). Consequently, they reduce the PZase-mediated conversion of PZA to its active form, POA. In our cohort, the MeltPro MTB/PZA assay detected pncA mutations in 44.5% (53/119) of RR-TB isolates, demonstrating strong agreement with WGS-defined genotypes (*κ* = 0.882). This consistency was slightly higher than that reported by Li et al. (2023). The difference likely stems from their use of sputum specimens, whereas our study used culture isolates, which provide more uniform and higher-quality DNA. Although population genetic backgrounds (Lineage 2 predominated in our cohort) could also contribute, further data are needed to explore this possibility. Notably, in this study, WGS confirmed that all PZA resistance-conferring mutations were located in the *pncA* gene, which likely contributed to the high concordance observed between the two methods. As shown in Table 2, there were 6 isolates that were WGS-positive but MeltPro-negative. According to the kit manufacturer’s explanation, this discrepancy may be attributed to heteroresistance—the coexistence of drug-susceptible and drug-resistant *M. tuberculosis* subpopulations within the same clinical specimen. Such heteroresistance can lead to signal interference in the MeltPro assay. This interpretation is supported by WGS, which detected heteroresistance in 5 of the 6 discordant isolates. These findings highlight the need for phenotypic drug susceptibility testing to resolve discordant results. Additionally, we observed that *pncA* mutations detected by the MeltPro assay were predominantly localized to the A-HEX, B-HEX, and C-ROX channels. However, because the commercial kit did not specify the nucleotide positions corresponding to individual channels, we could not delineate the exact base-level mutation sites in this study. While this limitation is acceptable for rapid clinical screening, it has two potential drawbacks. It may affect the ability to track specific mutation epidemiology, and it may hinder efforts to contribute data for refining genotype–phenotype correlations in databases.

The WGS results in this study revealed that point mutations in the *pncA* gene accounted for 67.2% of all detected cases, with 49 distinct mutation types identified. The most frequently mutated site was the −11 position in the promoter region, observed in 4 isolates (6.9%). This prevalence is slightly lower than the 7.9% reported in a nationwide multicenter study by Gao et al. (2020), but remains consistent with the 6.8% observed by Li et al. (2016) in strains from Zhejiang, Jiangsu, and Sichuan. Promoter region mutations are known to confer PZA resistance via downregulation of *pncA* expression, as demonstrated by Pang et al. (2017). Overall, the predominant mutation types in the *pncA* gene are largely consistent across different regions of China, though variations in mutation frequencies may occur due to differences in sample size, strain genetic diversity, or local medication practices.

We also assessed the phenotypic impact of structural mutations by comparing them with quantitative PZA MIC data. All five isolates carrying large deletions in *pncA* exhibited MICs ≥400 μg/mL, with four exceeding 800 μg/mL, suggesting loss of functional pyrazinamidase activity. Consistent with our findings, a recent large-scale genomic study by Kim et al. (2023) reported that among 10,725 *M. tuberculosis* isolates, 102 (1.0%) carried large deletions in *pncA*. All but two of these isolates were PZA-resistant. Notably, more than half of these *pncA* large deletion isolates (69/102, 67.6%) belonged to the East Asian lineage (Lineage 2). Interestingly, all five of our isolates harboring *pncA* large deletions also belonged to Lineage 2. This further supports the notion that this structural variant may be a genomic characteristic specific to the East Asian lineage. To the best of our knowledge, the study by Kim et al. (2023) was the first to demonstrate the frequent occurrence of *pncA* deletions in Lineage 2 M. tuberculosis isolates. Our findings provide independent confirmation of this association. Among the 14 isolates with frameshift mutations caused by small indels, 8 showed MICs ≥400 μg/mL. The remaining 5 showed no growth in positive control wells and were recorded as “NA” in the MIC assay. Under acidic conditions, these NA results likely reflect impaired bacterial fitness or a growth defect. This suggests that the mutations carried by these strains reduce their ability to survive or replicate in an acidic environment. Notably, no phenotypic heterogeneity was observed among independent isolates carrying the same frameshift mutation, including those with NA results. For mutations with interpretable MIC data (456delC and 495delC), the resistant phenotype was uniformly observed, suggesting a deterministic effect. The consistent NA outcomes for 389_390insA and 520_521insT further support that certain mutations compromise bacterial fitness under acidic conditions. However, the exact MIC levels could not be determined.

Regarding analytical methodology, two rounds of analysis using TB-Profiler yielded discrepant results for large deletions. This was likely attributable to updates in both the software and the WHO mutation catalogue between the two time points. Although their presence was ultimately confirmed, this discrepancy highlights the limitations of relying on a single analysis tool. As Hong et al. (2026) noted, integrating multiple mutation call sets may increase sensitivity. However, this comes at the cost of reduced specificity due to the inclusion of low-confidence associations. Moreover, as noted by Kim et al. (2023), the current WHO catalogue does not yet include large deletions, underscoring the need for a major update to formally incorporate them as resistance-conferring variants. This trade-off underscores the need for a balanced strategy. Such a strategy should prioritize cross-validated calls while exercising caution toward discordant outputs. Taken together, these findings provide direct experimental evidence linking *pncA* structural variants to elevated PZA MICs. This includes both large deletions and frameshift indels, supporting their classification as resistance-conferring mutations. The absence of phenotypic heterogeneity among independent isolates carrying identical frameshift mutations reinforces the reliability of WGS-based resistance prediction for such variants. It also provides further experimental validation for variants catalogued in existing molecular resistance databases.

This study has several limitations. First, growth failure occurred in nine PZA-free positive control wells in the BMD assay. This could be attributed to either technical factors—such as reduced bacterial viability due to prolonged storage or repeated subculturing—or biological factors. The latter are linked to specific mutations that impair bacterial fitness in an acidic environment. Distinguishing between these possibilities warrants further investigation. Second, all study isolates were collected from a single geographic region (Beijing) and a single year (2009). While this does not detract from the internal validity of our comparisons, it may limit the generalizability of the reported *pncA* mutation spectrum and resistance rates to other geographic settings or time periods. Third, the MeltPro MTB/PZA assay detects mutations only through predefined fluorescence channels and does not provide precise mutation site information, which limits the granularity of comparison with WGS data. Fourth, we acknowledge that the vast majority of isolates (95.0%) in this study belonged to Lineage 2, which limits the generalizability of our findings to settings with different lineage distributions, particularly Lineage 4-dominated regions. Finally, and most importantly, we did not perform PZA susceptibility testing using the MGIT 960 method, the current reference standard. This precludes a direct comparison between the BMD method and the reference standard and limits our ability to fully assess discrepancies between the two phenotypic methods. We plan to include MGIT 960 testing in future experiments to further explore the relationship between genotypic mutations and phenotypic drug susceptibility results. Specifically, we will focus on isolates carrying resistance mutations but with MICs below the critical concentration. We will employ multiple phenotypic and molecular methods for comprehensive validation. This will allow us to more fully evaluate their resistance characteristics and clinical implications.

In summary, the BMD test at a critical concentration of 200 μg/mL showed closer agreement with WGS-defined genotypes than the 100 μg/mL concentration in this dataset, and the MeltPro MTB/PZA assay demonstrated excellent agreement with WGS. However, we acknowledge that high agreement with WGS does not in itself constitute validation of phenotypic or clinical accuracy. Given the absence of an MGIT 960 comparator and the inherent uncertainties in WGS-based PZA resistance prediction—particularly for rare or structural variants—these findings should be interpreted as exploratory. Further studies incorporating phenotypic reference methods and clinical outcomes are needed to determine the optimal critical concentration and to establish the diagnostic role of rapid molecular assays for PZA susceptibility testing. Ongoing efforts to expand our cohort with more diverse strains and validate these findings in larger datasets may help inform future revisions of international guidelines for genotypic PZA resistance interpretation.

## Data Availability

The data presented in this study are deposited in the GSA (Genome Sequence Archive) repository, accession number CRA038525.

## Supplementary material

SUPPLEMENTARY FIGURE S1 Design of the MIC test plate for 19 anti-TB drugs.

SUPPLEMENTARY FIGURE S2 Changes in pH (before vs. after incubation).

SUPPLEMENTARY FIGURE S3 The mutation frequencies of each channel.

SUPPLEMENTARY TABLE S1 Summary of genomic accession numbers, pyrazinamide MICs, and MeltPro MTB/PZA assay results.

SUPPLEMENTARY TABLE S2 Genomic characteristics of large deletions confirmed in this study.

SUPPLEMENTARY TABLE S3 Indel and large deletion variants in pncA identified in this study and their corresponding PZA MICs.
