# Supplementary figures and images for: Toward precision detection of pyrazinamide resistance: critical concentration assessment and rapid molecular method validation
Source: Front Microbiol. 2026 May 25;17:1828630. doi: 10.3389/fmicb.2026.1828630 (PMC13243239; doi:10.3389/fmicb.2026.1828630)

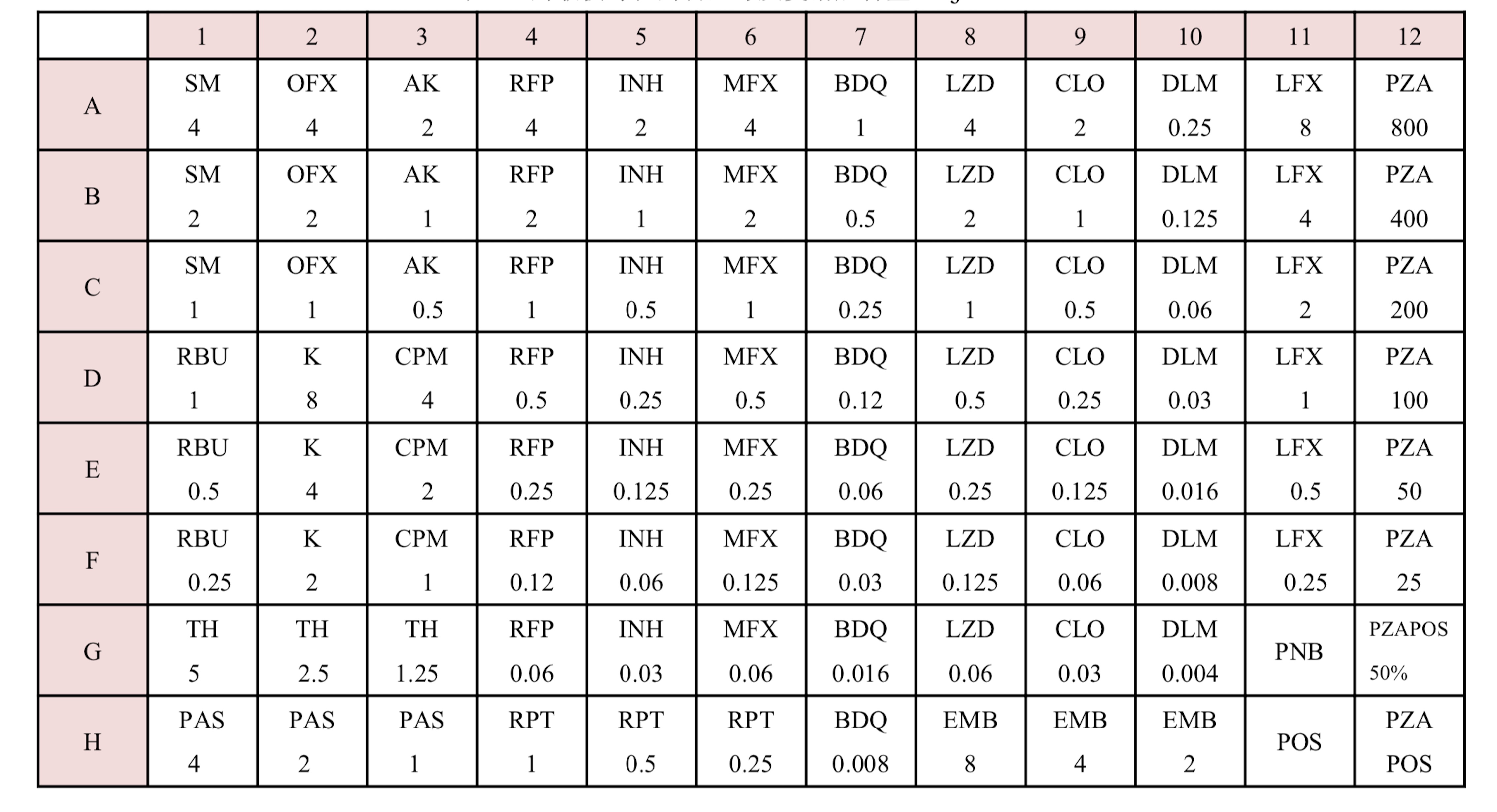

Supplement: SUPPLEMENTARY FIGURE S1 — Design of the MIC test plate for 19 anti-TB drugs. [file Image_1.JPEG]

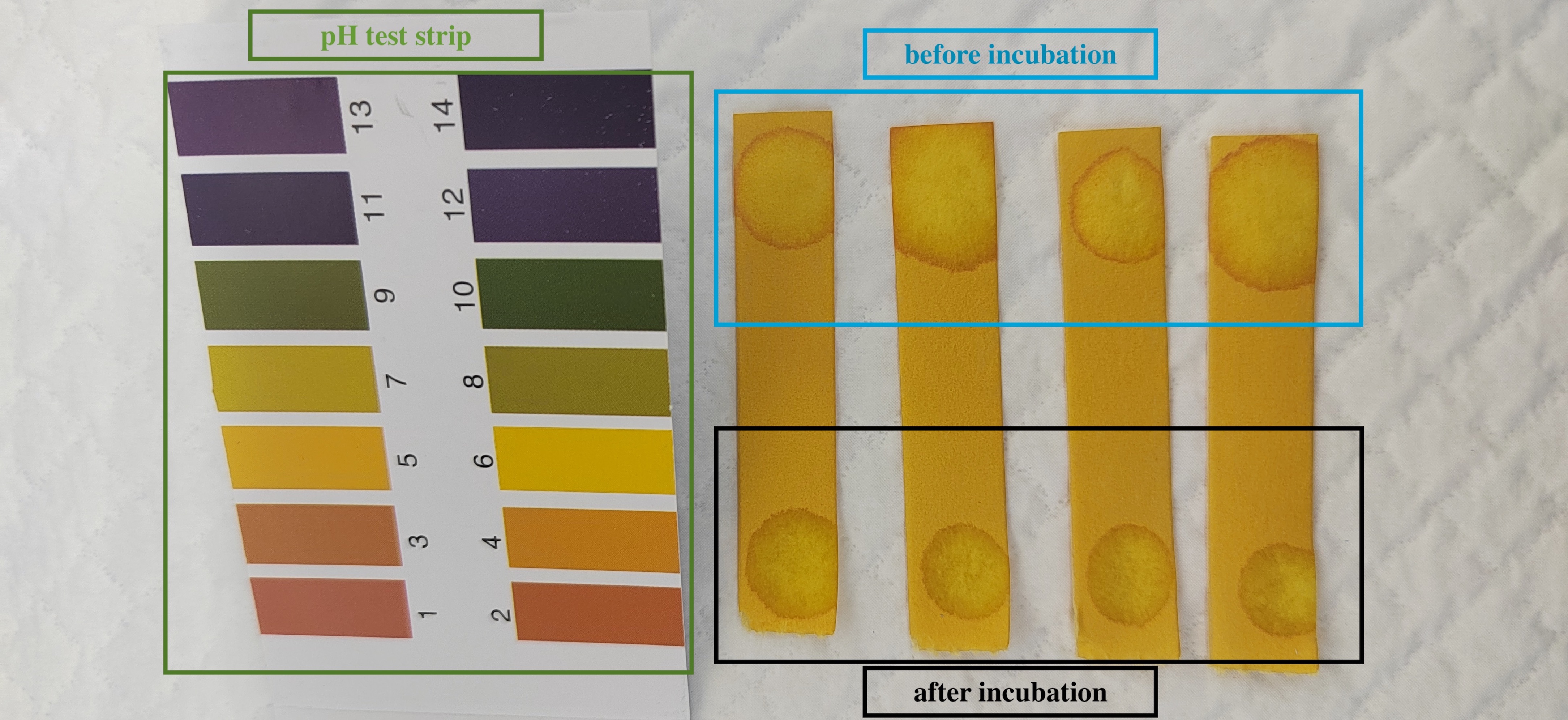

Supplement: SUPPLEMENTARY FIGURE S2 — Changes in pH (before vs. after incubation). [file Image_2.JPEG]

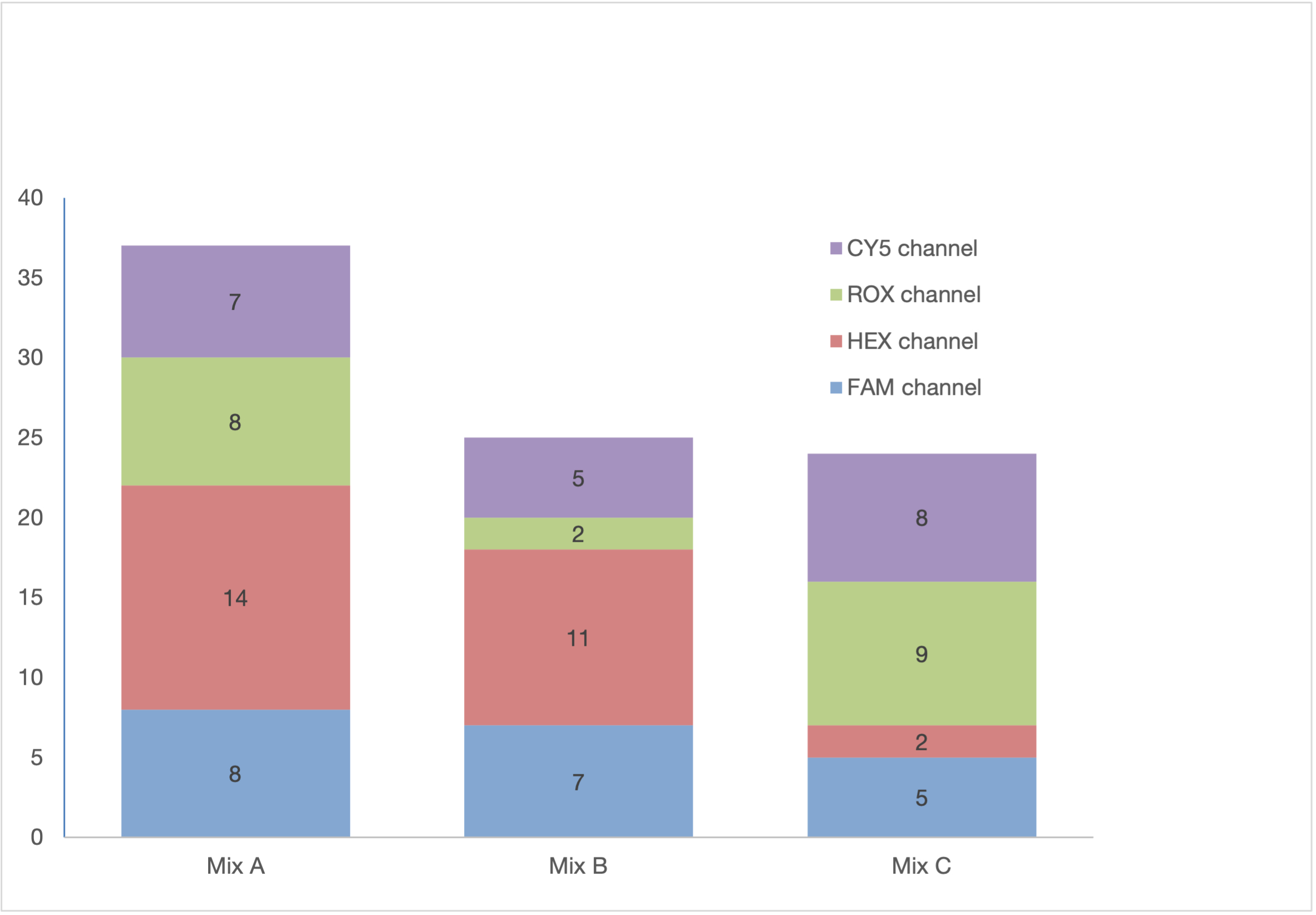

Supplement: SUPPLEMENTARY FIGURE S3 — The mutation frequencies of each channel. [file Image_3.JPEG]
