# Supplementary material for: Toward precision detection of pyrazinamide resistance: critical concentration assessment and rapid molecular method validation
Source: Front Microbiol. 2026 May 25;17:1828630. doi: 10.3389/fmicb.2026.1828630 (PMC13243239; doi:10.3389/fmicb.2026.1828630)
Supplement: SUPPLEMENTARY TABLE S3 — Indel and large deletion variants in pncA identified in this study and their corresponding PZA MICs. [file Table_3.DOCX]

Supplementary Material

## Supplementary Table S3

| ***pncA* Variant Category** | **Site and Nucleic change** | **No. of Isolates** | **sample ID** | **PZA MIC(µg/mL)** |
| --- | --- | --- | --- | --- |
| Deletion |  | **7** |  |  |
|  | 116delC | 1 | 09-702 | 800 |
|  | 366delA | 1 | 09-367 | NA |
|  | 399delT | 1 | 09-969 | 800 |
|  | 456delC | 2 | 09-1502 | 800 |
|  |  |  | 09-245 | 800 |
|  | 495delC | 2 | 09-1669 | 400 |
|  |  |  | 09-272 | 800 |
| Insertion/duplication |  | **7** |  |  |
|  | 16_17insC | 1 | 09-1465 | 800 |
|  | 389_390insA | 2 | 09-212 | NA |
|  |  |  | 09-362 | NA |
|  | 520_521insT | 2 | 09-151 | NA |
|  |  |  | 09-289 | NA |
|  | 284dupA | 1 | 09-1168 | 50 |
|  | 409dupC | 1 | 09-757 | 800 |
| Large deletion |  | **5** |  |  |
|  | 270_*2851del | 1 | 09-1210 | 800 |
|  | 277_427del | 1 | 09-700 | 800 |
|  | 291_*5011del | 1 | 09-936 | 800 |
|  | 371_*857 del | 1 | 09-1594 | 400 |
|  | 515_*66 del | 1 | 09-589 | 800 |

Note: Not Available(NA)

**Supplementary Table S3.** Indel and large deletion variants in pncA identified in this study and their corresponding PZA MICs.
